# Advances in real-time magnetic resonance imaging of the vocal tract for speech science and technology research

**DOI:** 10.1017/ATSIP.2016.5

**Published:** 2016-03-31

**Authors:** ASTERIOS TOUTIOS, SHRIKANTH S. NARAYANAN

**Affiliations:** Signal Analysis and Interpretation Laboratory (SAIL), University of Southern California (USC), 3740 McClintock Avenue, Los Angeles, CA 90089, USA

**Keywords:** Speech production, Real-time MRI, Vocal tract shaping, Articulation

## Abstract

Real-time magnetic resonance imaging (rtMRI) of the moving vocal tract during running speech production is an important emerging tool for speech production research providing dynamic information of a speaker's upper airway from the entire mid-sagittal plane or any other scan plane of interest. There have been several advances in the development of speech rtMRI and corresponding analysis tools, and their application to domains such as phonetics and phonological theory, articulatory modeling, and speaker characterization. An important recent development has been the open release of a database that includes speech rtMRI data from five male and five female speakers of American English each producing 460 phonetically balanced sentences. The purpose of the present paper is to give an overview and outlook of the advances in rtMRI as a tool for speech research and technology development.

## I. INTRODUCTION

A long-standing challenge in speech research is obtaining accurate information about the movement and shaping of the vocal tract. Dynamic vocal tract-imaging data are crucial for investigations into phonetics and phonological theory, where they afford insights into the nature and execution of speech production goals, the relationship between speech articulation and acoustics, and the mechanisms of speech motor control. Such data are also important for advancing the knowledge and treatment of speech pathologies, and to inform models used in speech technology applications, such as machine speech recognition and synthesis.

A number of techniques are available for the acquisition of data on the kinematics of speech production. Electromagnetic articulography (EMA) [1] uses electromagnetic fields to track the positions of markers attached on articulators in two or three dimensions with sampling rates up to 400 Hz. X-ray microbeam (XRMB) [2] generates a very narrow beam of high-energy X-ray, and rapidly directs this beam, under high-speed computer control, to track the motions of 2–3 mm diameter gold pellets glued to articulators with rates up to 160 Hz. Electropalatography (EPG) [3] uses an artificial palate with embedded electrodes to record linguopalatal patterns of contact, typically at 100–200 Hz. Ultrasound [4, 5] can be used to image the tongue, and X-ray [6–9] or videofluoroscopy [10] to image the sagittal projection of the entire vocal tract at frame rates typically between 10 and 50 Hz. Synchronized-sampling (repetitive) MRI can be used to reconstruct tongue motion in two-dimensional (2D) or 3D from multiple repetitions of an utterance [11, 12].

Nevertheless, it is still difficult to safely obtain information about the location and movement of speech articulators in all parts of the vocal tract (like the tongue, velum, and larynx, hidden from plain view) and at sufficiently high sampling rates with respect to their natural movement speed during speech. All aforementioned speech production data acquisition technologies are limited in one sense or the other. EMA and XRMB both provide rich data about the movement of sensors or markers attached on lingual and labial fleshpoints, but such sensors/markers cannot be easily placed at posterior locations on the tongue, on the velum, in the pharynx, or in the larynx; hence these technologies are limited in terms of the spatial coverage of the complex vocal tract geometry. EPG is restricted to contact measurements at the palate. Ultrasound cannot consistently or reliably image the tongue tip, the pharyngeal surface of the tongue (because of the obscuring effect of the hyoid bone), or the opposing surfaces such as the hard and soft palate (and hence the airway shaping). X-ray and videofluoroscopy expose subjects to unacceptable levels of radiation. In early development, magnetic resonance imaging (MRI) has been used to capture images of static configurations of the vocal tract, but while subjects sustain continuant speech sounds over unnaturally long periods of time. In synchronized-sampling cine-MRI (or repetitive cine-MRI), articulatory dynamics of connected speech may be reconstructed from large numbers of repetitions (which should be identical) of short utterances.

Real-time magnetic resonance imaging (rtMRI) is an important emerging tool for speech production research [13, 14], providing dynamic information from the entire midsagittal plane of a speaker's upper airway, or any other scan plane of interest, from arbitrary, continuous utterances with no need of repetitions. Midsagittal rtMRI captures not only lingual, labial, and jaw motion, but also articulation of the velum, pharynx and larynx, and structures such as the palate and pharyngeal wall – regions of the tract that cannot be easily or well observed using other techniques. While sampling rates are currently lower than for EMA or XRMB, rtMRI is a unique source of dynamic information about vocal tract shaping and global articulatory coordination. Because rtMRI allows unparalleled views of the state of articulation in regions of the tract from which it has previously proven problematic to obtain accurate data, this technique is beginning to offer new insights into the the goals of production of coronal [15], pharyngeal [16] and nasal [17] segments, and the coordination of articulators during the production of multi-gestural segments in speech [18–20]. Most importantly, rtMRI data also provide a rich source of information about articulation in connected speech, which is proving to be valuable in the refinement of existing speech models and the development of new models of representation for automatic speech recognition (ASR) and other speech processing applications. RtMRI of the upper airway (a definition that also includes studies of other functions of the vocal tract besides speech, such as swallowing) is an actively growing research area [21–27].

**Fig. 1:**
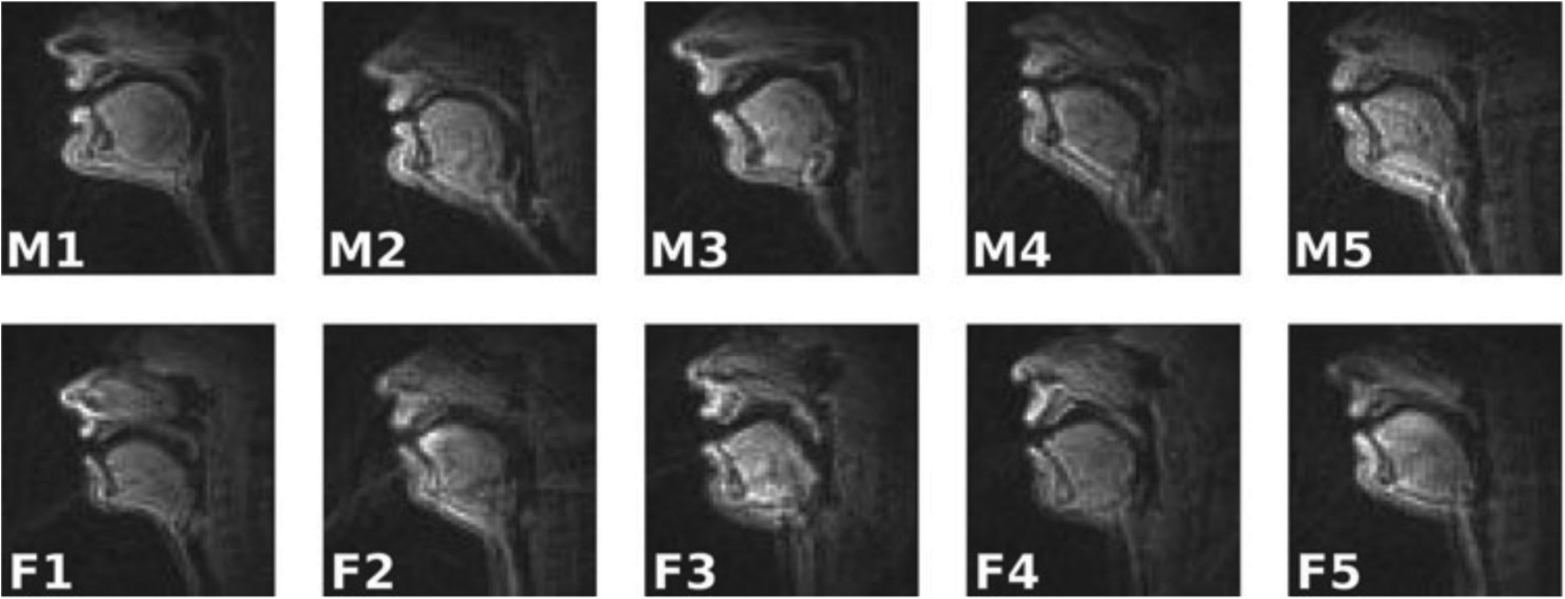
Example rtMRI frames from the ten speakers in the USC-TIMIT database (top row, male; bottom row, female).

**Fig. 2:**
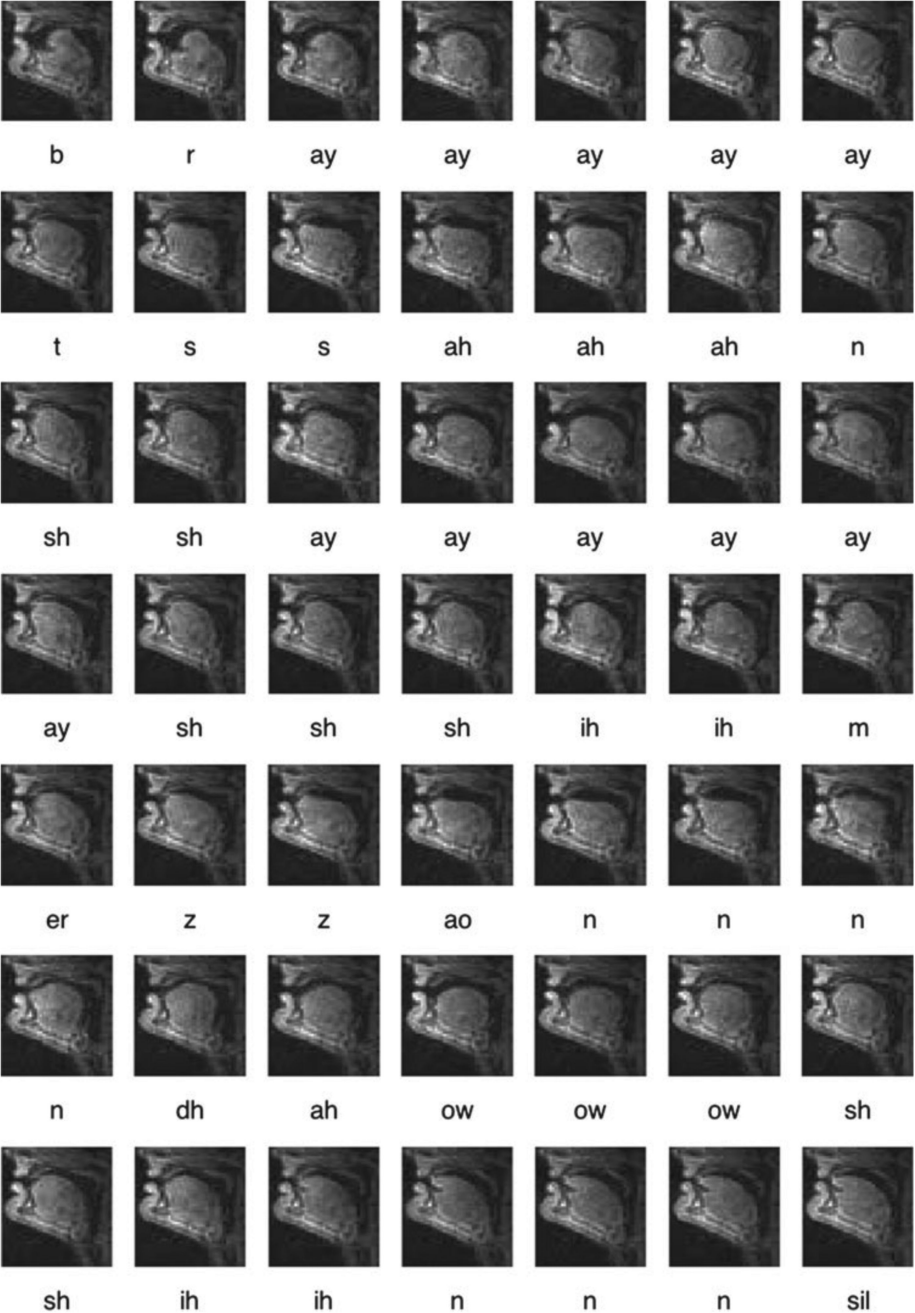
Example rtMRI sequence from the USC-TIMIT database. A male subject utters the sentence “Bright sunshine shimmers on the ocean” (one of the 460 MOCHA-TIMIT sentences included for each subject). Note that there is a zoom into the frames, as compared to Fig. 1. The phonetic labels are a result of automatic alignment. The symbol “sp” stands for “space” and “sil” for “silence”.

The present paper provides an overview of rtMRI for speech research that is particularly being developed by an interdisciplinary team at the University of Southern California (USC). It summarizes their advances in creating and refining rtMRI acquisition methods, developing analysis tools, collecting multilingual speech and vocal production data, and using them to address scientific and technology problems of interest. This includes the public release of a unique corpus of articulatory data, called the USC-TIMIT database [28], available from http://sail.usc.edu/span/usctimit/, which includes rtMRI data from ten speakers, each uttering the same 460 sentences used in the context of the popular MOCHA-TIMIT database [29] of EMA, EPG, and electroglottographic (EGG [30]) data. This set of sentences was designed to elicit all phonemes of English in a wide range of prosodic and phonological contexts, with the connected speech processes characteristic of spoken English, including assimilations, lenitions, deletions, and mergers. USC-TIMIT also includes EMA data collected separately from four of the subjects. See Figs 1 and 2 for example images from the database.

The rest of this paper elaborates on some technical aspects of rtMRI data acquisition at USC (Section II); describes associated tools for data analysis (Section III); reviews illustrative applications (Section IV), and discusses challenges and future directions (Section V).

## II. DATA ACQUISITION

The first two subsections of this section briefly discuss some technical details of the acquisition and reconstruction protocols that have been used most extensively at USC, including for the USC-TIMIT corpus. The third subsection discusses some alternative protocols and recent developments. Note that several details are shared among protocols. This will be implied unless otherwise noted.

### A) Imaging

The upper airways of the subjects are imaged while they lay supine in the MRI scanner. Subjects have their heads firmly but comfortably padded at the temples to minimize motion of the head. Stimuli are presented in large text on a back-projection screen, from which subjects can read from inside the scanner bore without moving their head. The nature of the experiment and the protocol is explained to subjects before they enter the scanner, and subjects are paid for their time upon completion of the session. The overall recording time for each subject includes calibration and breaks in-between stimuli. The USC Institutional Review Board has previously approved the data collection procedures.

Data are acquired at Los Angeles County Hospital on a Signa Excite HD 1.5T scanner (GE Healthcare, Waukesha, WI) with gradients capable of 40 mT/m amplitude and 150 mT/m/ms slew rate. A body coil is used for radio frequency (RF) signal transmission. A custom upper airway receiver coil array is used for RF signal reception. This four-channel array includes two anterior coil elements and two coil elements posterior to the head and neck. However, only the two anterior coils are used for data acquisition. The posterior coils are not used because they have been previously shown to result in aliasing artifacts.

The rtMRI acquisition protocol is based on a spiral fast gradient echo sequence. This is a scheme for sampling the spatial frequency domain (*k*-space) in which data are acquired in spiraling patterns. Thirteen interleaved spirals together form a single image. Each spiral is acquired over 6.164 ms (repetition time (TR), which includes slice excitation, readout, and gradient spoiler) and thus every image comprises information spanning 13 × 6.164 = 80.132 ms. A sliding window technique is used to allow for view sharing and thus increase frame rate [13]. The TR-increment for view sharing is seven acquisitions, which results in the generation of an MRI movie with a frame rate of 1/(7 × TR) = 1/ (7 × 6.164 ms) = 23.18 frames/s [13, 14, 31].

The imaging field of view is 200 mm × 200 mm, the flip angle is 15°, and the receiver bandwidth ±125 kHz. Slice thickness is 5 mm, located midsagittally; image resolution in the sagittal plane is 68 × 68 pixels (2.9 mm × 2.9 mm). Scan plane localization of the midsagittal slice is×performed using RTHawk (HeartVista, Inc., Los Altos, CA), a custom real-time imaging platform [32].

MR image reconstruction is performed using MATLAB (Mathworks, South Natick, MA). Images from each of the two anterior coils of the four-channel coil array are formed using gridding reconstruction [14, 33]; and the two images are combined by taking their root sum-of-squares in order to improve image signal-to-noise ratio (SNR) and spatial coverage of the vocal tract.

### B) Audio acquisition

Acquiring and synchronizing the acoustic signal with the MRI data – which is crucial in order to facilitate the interpretation and analysis of the articulatory information in the speech production videos – presents numerous technical challenges. In the deployed system, audio is simultaneously recorded at a sampling frequency of 20 kHz inside the MRI scanner while subjects are imaged, using a fiber-optic microphone (Optoacoustics Ltd., Moshav Mazor, Israel) and custom recording and synchronization setup. The audio signal is controlled through the use of a sample clock derived from the scanner's 10 MHz master clock, and triggered using the scanner RF master-exciter un-blank signal, which is a TTL (Transistor–Transistor Logic) signal synchronous to the RF pulse.

**Fig. 3:**
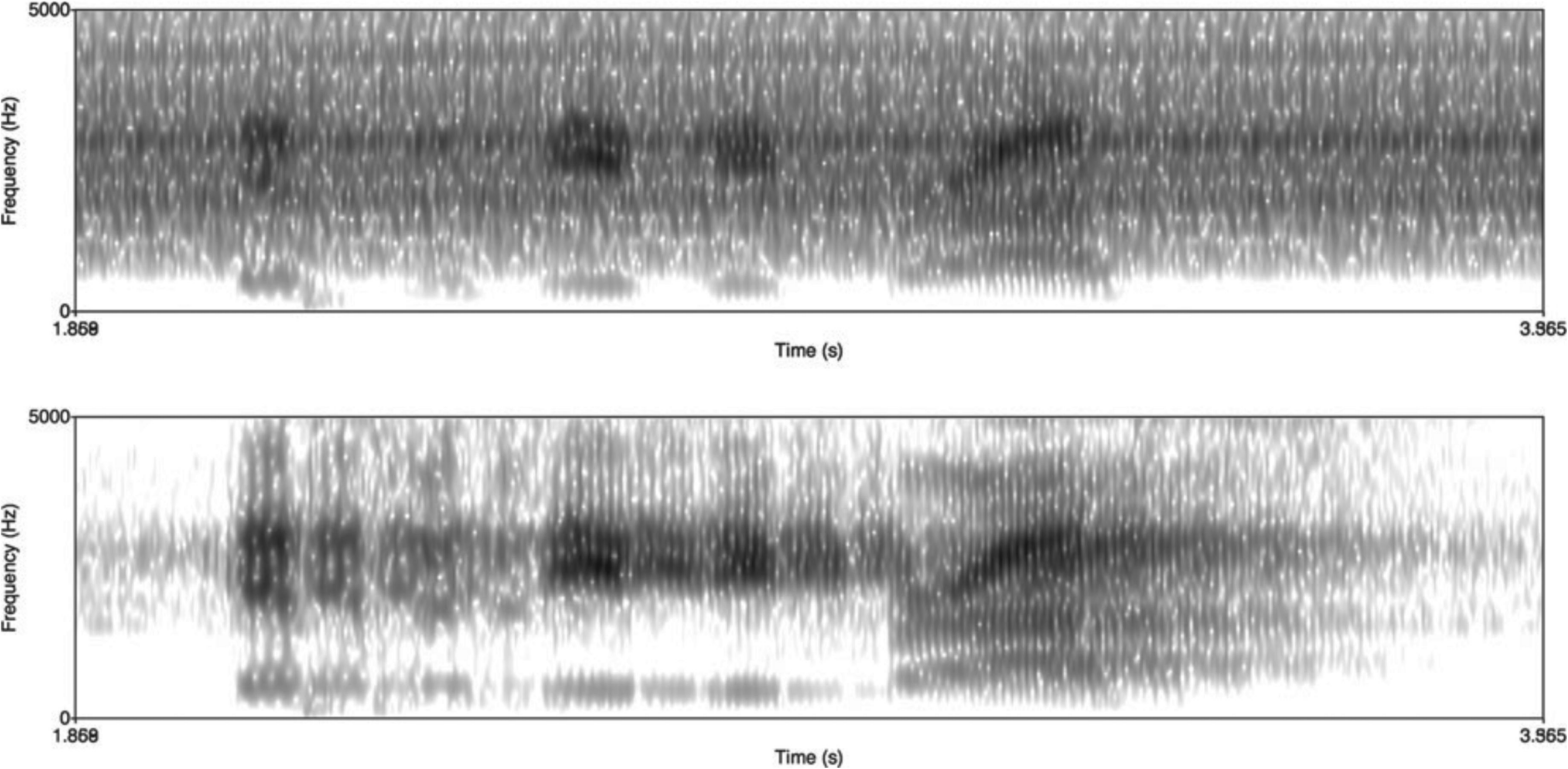
Spectrograms of the audio, recorded concurrently with the rtMRI data, for the utterance “This was easy for us” spoken by a female subject before (top) and after (bottom) de-noising.

Apart from synchronization, another challenge to acquiring good quality audio is the high noise level generated by the operation of the MRI scanner. It is important that this noise be canceled satisfactorily in order to perform further detailed analyses of the audio for linguistic and statistical modeling purposes. For the sequences in Table 1, the MRI noise has a specific periodic structure, which enables noise cancellation using a custom adaptive signal processing algorithm which exactly takes into account this periodic structure [34]. See Fig. 3 for an example of noise cancellation.

Note that subjects wear earplugs for protection from the scanner noise, but are still able to hear loud conversation in the scanner room and to communicate effectively with the experimenters via both the fiber-optic microphone setup as well as the in-scanner intercom system.

### C) Alternative protocols

Three more rtMRI acquisition protocols based on spiral fast gradient echo sequences have been extensively used, according to the purpose of the specific experiment. The technical details of the sequences employed are summarized in Table 1. Sequence 1 in the table is the one described in the previous subsections. Sequences 2 and 3, like Sequence 1, make use of the four-channel coil array already discussed. The more recent Sequence 4 makes use of an eight-channel array that has four elements on either side of the jaw. Sequence 4 combines fast spirals with sparse sampling and constrained reconstruction, enabling frame rates of up to 83-frames/s and multi-slice imaging [35].

Sequence 1 is the most efficient in terms of SNR, i.e. it provides clearer images than, at least, Sequences 2 and 3. The SNR of Sequence 4 is very difficult to quantify, as this is coupled with constrained reconstruction through a nonlinear process. Visual inspection of data collected with Sequence 4 shows no degradation of the image quality compared with Sequence 1. Imaging of the area around the glottis is improved as a result of the eight-channel coil array configuration.

Audio de-noising for Sequences 2 and 3 is done using the same method as that for Sequence 1. However, Sequence 4 does not exhibit the same periodic structure as the other sequences. To achieve its de-noising, an audio enhancement method using dictionary learning and wavelet packet analysis that does not rely on periodicity has been recently developed [36].

We finally note that the USC team has also developed a protocol for accelerated static volumetric upper-airway MRI acquisition, which captures the 3D volume of the upper airway in as fast as 7 s [37, 38]. This has enabled capturing the 3D articulation of the full set of continuant English phonemes from several subjects, with no particular difficulty in sustaining the speech sounds for the required amount of time.

## III. DATA ANALYSIS TOOLS

While some speech production phenomena may be studied by manually inspecting the raw rtMRI data and measuring the timing of articulatory events identified in these image sequences [e.g., 19], many other aspects of speech production require additional signal processing and analysis. A number of tools to aid inspection and analysis of rtMRI data have been developed at USC.

### A) Data inspection and labeling

**Fig. 4:**
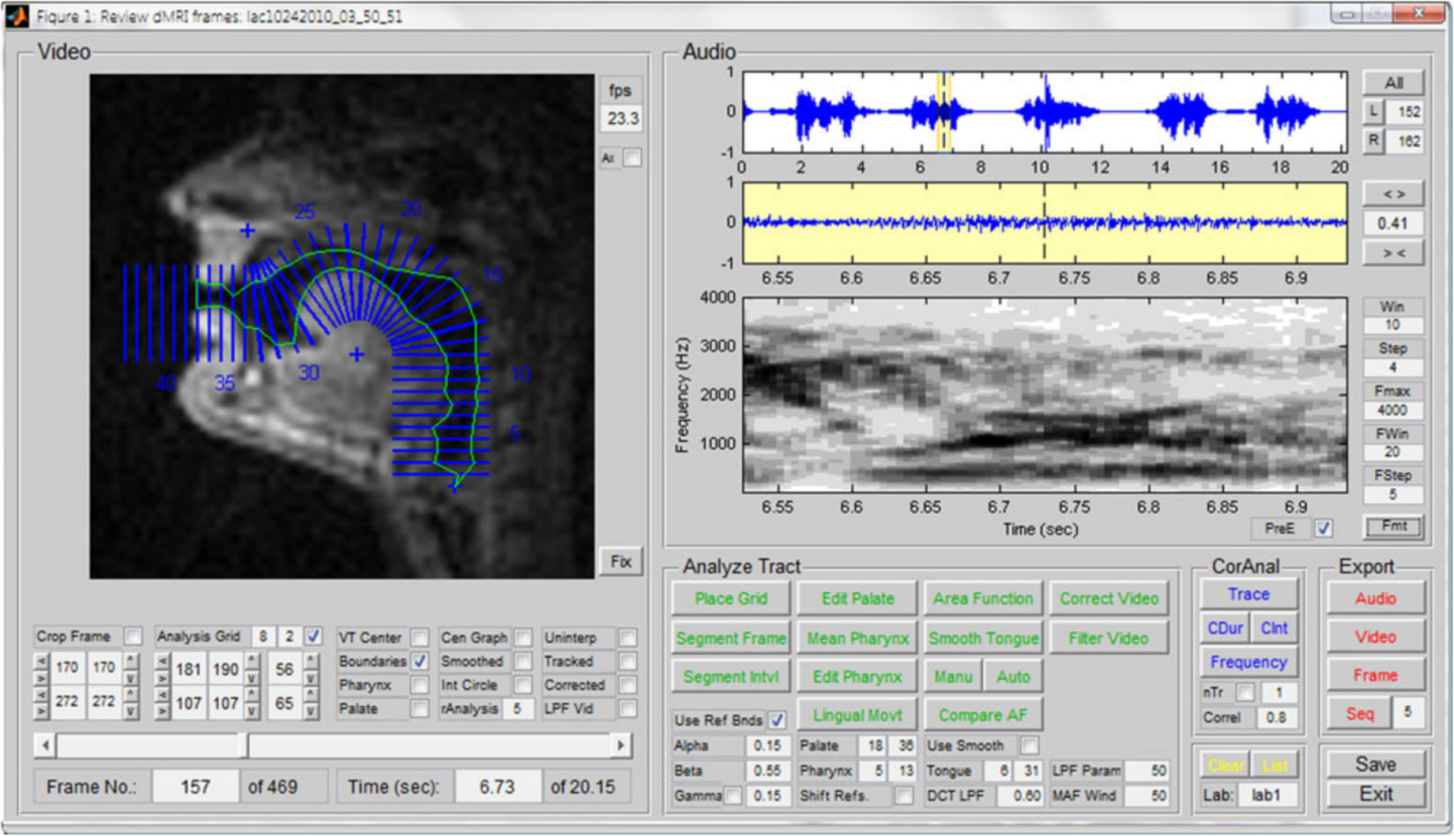
GUI allowing for audition, labeling, tissue segmentation, and acoustic analysis of the rtMRI data, displaying an example of parametric segmentation.

A graphical user interface (GUI) has been developed to allow for audition, labeling, tissue segmentation, and acoustic analysis of rtMRI data. The primary purpose of this tool is to allow users to browse the database frame-by-frame, inspect synchronized audio and video segments in real time or at slower frame rates, and label speech segments of interest for further analysis with the supporting tool set. The GUI also facilitates automatic formant and pitch tracking, and rapid semi-automatic segmentation of the upper airway in sequences of video frames, for visualization of tongue movement, or as a precursor to dynamic parametric analysis of vocal tract shaping. Fig. 4 shows a screenshot of this GUI.

### B) Automatic articulator tracking

By identifying air-tissue boundaries in rtMRI images, the position and configuration of articulators can be compared at different points in time. Vocal tract cross-distances may also be calculated, and changes in lingual posture can be examined during the production of different speech segments. For many types of speech, vocal tract outlines may be tracked using semi-automatic or fully automatic identification of tissue boundaries in rtMRI data.

**Fig. 5:**
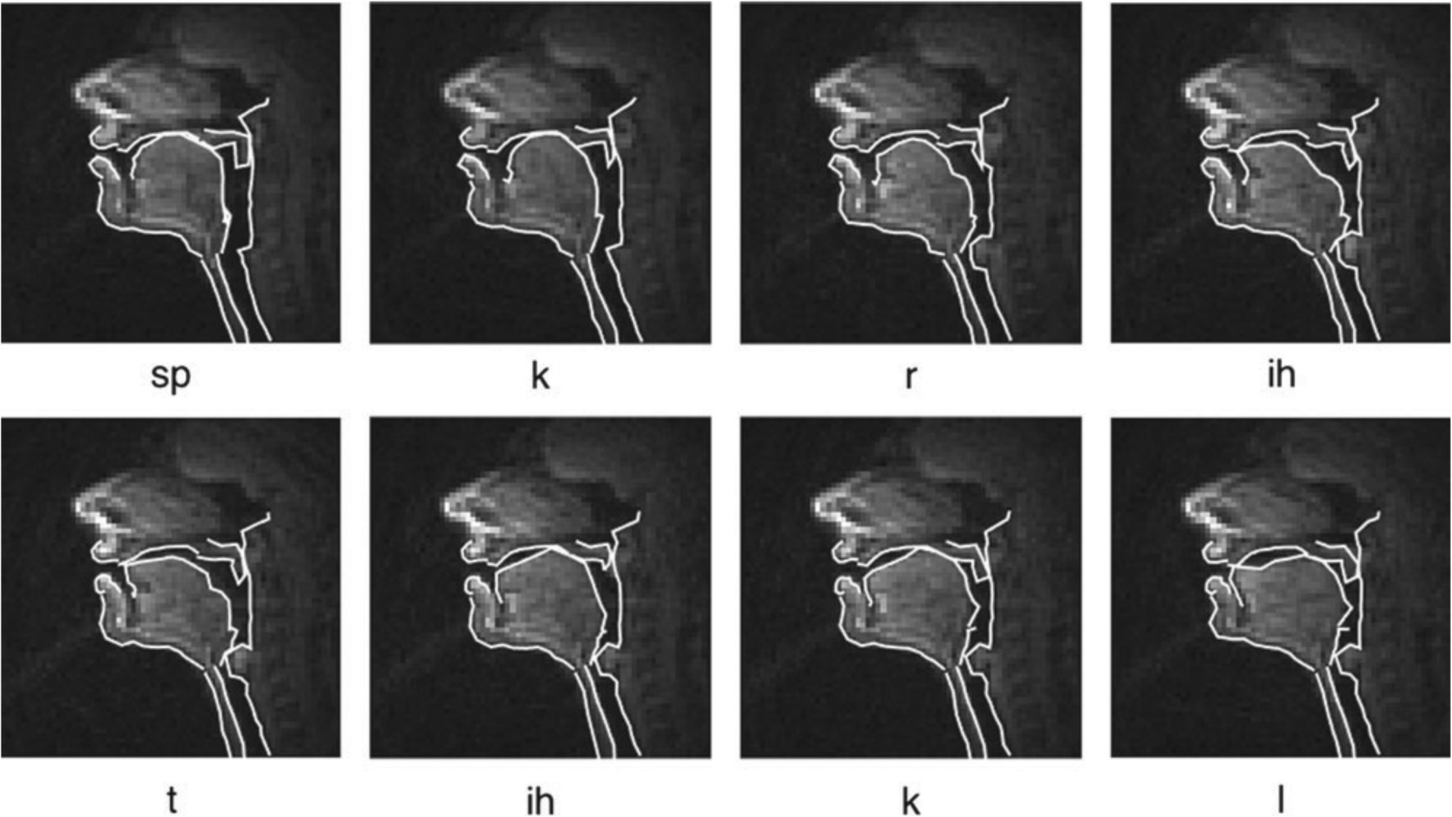
Example of region segmentation (white outlines) of articulators in rtMRI data. The word uttered by the female subject is “critical”. The symbol “s” stands for “space”.

Unsupervised segmentation of regions corresponding to the mandibular, maxillary, and posterior areas of the upper airway has been achieved by exploiting spatial representations of these regions in the frequency domain, the native domain of MRI data [39]. The segmentation algorithm uses an anatomically informed object model, and returns a set of tissue boundaries for each frame of interest, allowing for quantification of articulator movement and vocal tract aperture in the midsagittal plane. The method makes use of alternate gradient vector flows, non-linear least-squares optimization, and hierarchically optimized gradient descent procedures to refine estimates of tissue locations in the vocal tract. Thus, the method is automatic and well suited for processing long sequences of MR images. Fig. 5 shows an example of air-tissue boundaries produced by this algorithm. Obtaining such vocal tract contours enables the calculation of vocal-tract midsagittal cross-distances, which in turn can be used to estimate area functions, via standard reference sagittal-to-area transformations [40–42].

The above segmentation method requires significant computational resources. As a faster (yet less accurate) alternative, a method of rapid semi-automatic segmentation of rtMRI data for parametric analysis has been developed, which seeks pixel intensity thresholds distributed along tract-normal grid-lines and defines airway contours constrained with respect to a tract centerline constructed between the glottis and lips [43, 44]. A version of this method has been integrated in the aforementioned GUI (see Fig. 4).

An optional pre-processing step before the application of these segmentation algorithms is the correction of any brightness gradient in the rtMRI sequences, which is a result of the coil configuration. To this end, a thin-plate spline-based intensity correction procedure [45] is applied, to obtain an estimate of the combined coil sensitivity map, which is constant for all images contained in the sequence. Thus, corrected maximally flat magnitude images can be obtained [39].

### C) Direct image analysis

While boundary detection is important for capturing the posture of individual articulators at different points in time, it is often enough to observe the dynamics of the formation and release of constrictions in different regions of the vocal tract [46]. Pixel intensity in an MR image is indicative of the presence or absence of soft tissue; as a result, tissue movement into and out of a region of interest in the upper airway may be estimated by calculating the change in mean pixel intensity in the vicinity of that region. Using this concept, a direct image analysis method has been developed that by-passes the need to first identify tissue boundaries in the upper airway [47, 48]. Constriction location targets may be automatically estimated by identifying regions of maximally dynamic correlated pixel activity along the palate and at the lips, and closure and release gesture timings may be estimated from landmarks in the velocity profile derived from the smoothed intensity function [49].

## IV. APPLICATIONS

The capability of vocal tract rtMRI data acquisition creates research opportunities for new and deeper insights in a number of areas. The promise held by these data and methods has already begun to be realized in a number of domains, from phonetics and phonological theory research to speech technology research. In this section, some findings of the USC team and applications that showcase the utility of rtMRI as an emerging tool for speech research are briefly summarized.

### A) Compositionality of speech production

The USC team has been combining the rtMRI technology with linguistically informed analysis of vocal tract constriction actions in order to investigate the production and cognitive control of the compositional action units of spoken language. Of particular interest is the framework of Articulatory Phonology [50], which provides a theoretical foundation for the team's work. Note that this effort has required the collection of specifically tailored rtMRI data, besides general-purpose data, such as those of the USC-TIMIT database.

Speech is dynamic in nature: it is realized through time-varying changes in vocal tract shaping, which emerge lawfully from the combined effects of multiple constriction events distributed over space (i.e. subparts of the vocal tract) and over time. Understanding this dynamic aspect is fundamental to linguistic studies and is intended through the USC team's research to be added to the fields current – essentially static – approach to describing speech production.

RtMRI allows pursuing such a goal through examining the decomposition of speech into such cognitively controlled vocal tract constriction events, or gestures. Of specific interest are: (i) the compositionality in space, i.e. the deployment of concurrent gestures distributed spatially, over distinct constriction effectors within the vocal tract; (ii) the compositionality in time, i.e. the deployment of gestures temporally; and (iii) the characterization of articulatory setting, i.e. the set of postural configurations that the vocal tract articulators tend to be deployed from and return to in the process of producing fluent and natural speech.

An example study on the compositionality of speech production in space examined retroflex stops and rhotics in Tamil [51]. The study revealed that in some contexts these consonants may be achieved with little or no retroflexion of the tongue tip. Rather, maneuvering and shaping of the tongue in order to achieve post-alveolar contact varies across vowel contexts. Between back vowels /a/ and /u/, post-alveolar constriction involves curling back of the tongue tip, but in the context of the high front vowel /i/, the same constriction is achieved by tongue bunching. Results supported the notion that so-called retroflex consonants have a specified target constriction in the post-alveolar region, but that the specific articulations employed to achieve this constriction are not fixed.

An example line of research on the compositionality in time examined the coordination of velic and oral gestures for nasal consonants. For English /n/ [18], it was found that near-synchrony of velum lowering and tongue tip raising characterizes the timing for onsets, while temporal lag between the gestures is characteristic for codas, supporting and extending previous findings for /m/ [52]. In French, which, unlike English, uses nasal vowels, the coordination of velic and oral gestures was found to be more tightly controlled, to allow the distinction between nasal vowels and consonants [17]. But, while the nature of the coordinative relation was different between French and English, the timing of the corresponding gestures varied in the same way as a function of prosodic context.

Regarding the characterization of articulatory setting, research at USC supported the hypothesis that pauses at major syntactic boundaries (i.e. grammatical pauses), but not ungrammatical (e.g. word search) pauses, are planned by a high-level cognitive mechanism that also controls the rate of articulation around these junctures [53]. The hypothesis was that postures adopted during grammatical pauses in speech are more mechanically advantageous compared to postures assumed at absolute rest, i.e. that equal changes in articulatory posture result to greater changes in the space of speech tasks. This hypothesis was verified using locally weighted linear regression to estimate the forward map from low-level articulator variables to high-level task variables [54]. The analysis showed that postures assumed during grammatical pauses in speech, as well as speech-ready postures, are significantly more mechanically advantageous than postures assumed during absolute rest.

### B) Speaker specificity

Speakers have diverse vocal-tract morphologies, which affect their speech production (note, for example, the different vocal tracts of the ten USC-TIMIT speakers in Fig. 1). The USC team has started using rtMRI data, collected from diverse speakers, to study how individual vocal morphological differences are reflected in the acoustic speech signal and what articulatory strategies are adopted in the presence of such morphological differences to achieve speech invariance, either perceptual or acoustic. The capability of the USC team to collect large volumes of data from diverse speakers is crucial to this effort.

Initial work with rtMRI has focused on individual differences in the size, shape, and relative proportions of the hard palate and posterior pharyngeal wall. Specific aims were: to characterize such differences [55]; to examine how they relate to speaker-specific articulatory and acoustic patterns [56]; and to explore the possibility of predicting them automatically from the acoustic signal [57].

The long-term objective of this ongoing work is to improve scientific understanding of how vocal-tract morphology and speech articulation interplay and explain the variant and invariant aspects of speech signal properties within and across talkers.

This line of research may benefit automatic speaker recognition technology. State-of-the-art automatic speaker-recognition methods yield strong results over a range of read and spontaneous speech domains, utterance lengths, and noise conditions [58–60]. In several studies, the technology performs better than even trained human listeners [61]. Despite considerable success in automatic speaker recognition, technologies are not informative about articulatory differences between speakers. RtMRI data can be used to improve the interpretability of such systems by associating acoustic differences to articulatory ones [62].

### C) Articulatory-acoustic maps

Benefits from rtMRI are also expected in the context of studying the forward map from articulation to acoustics (or, articulatory synthesis) and the inverse (acoustic-to-articulatory) mapping. Note that these problems have been classically addressed without taking into account speaker variability.

Characterizing the many-to-one mapping from representations in the articulatory space to those in the acoustic space is a central problem in phonological theory [63, 64]. The problem is compounded by our incomplete knowledge of the articulatory goals of production. Data from rtMRI provide a rich new source of information, which can inform research in this domain. This, in turn, can simplify the modeling of the articulatory-acoustic map and lead to more accurate estimates of articulatory features from the acoustic signal in acoustic-to-articulatory inversion. Since rtMRI provides rich information of the speech production process, an analysis of the non-uniqueness in articulatory-to-acoustic mappings using various rtMRI derived features can be performed to provide insight into the relationship between various articulatory features and the non-uniqueness in the mapping.

An important tool to be in place in order to achieve the above research goals is an articulatory synthesizer, i.e. a simulation of the articulatory-to-acoustic relationship in the vocal tract. Work has been done using Maeda's time-domain vocal tract simulation [65] to synthesize speech on the basis of EMA data [66], with the full midsagittal vocal tract profile being inferred from EMA using Maeda's articulatory model [40]. RtMRI, on the other hand, readily provides (i.e. after segmentation described in [39]) the full midsagittal profiles, and ongoing work aims at using rtMRI information for articulatory synthesis. Note that the synthesizer addresses the problem of synthesizing running (co-articulated) speech, and can be adapted to reflect different vocal-tract morphologies.

### D) The potential for ASR

Dynamic articulatory data have the potential to inform approaches to ASR [67, 68]. Since it provides such a rich source of global information about vocal tract dynamics during speech production, the discriminatory power of rtMRI-derived production features may help realize this potential in ASR. Additionally, examining the extent to which production-oriented features can provide information complementary to that provided by acoustic features can offer further insights into the role of articulatory knowledge in ASR [69, 70].

From a more theoretical viewpoint, there have been several well-known hypotheses regarding the relation between production and perception systems in human speech communication [71, 72]. Quantitatively modeling these relationships in order to develop better models of automatic speech and speaker recognition is a very challenging task that can benefit vastly from the availability of rich speech production data. For example, using mutual information as a metric, it has been shown in a data-driven manner that the nonuniform auditory filterbank in the human ear (receiver) is optimal in providing least uncertainty in decoding articulatory movements in the human speech production system (transmitter) [73]. This finding indicates that the design of the filterbank for speech recognition systems needs to be optimally designed with respect to the characteristics of the speech production system.

More such computational models need to be developed in order to understand the effect of speaker dependence, language effect, pathologies and paralinguistic features in speech and speaker recognition tasks, particularly to discover robust recognition models. RtMRI data may be central to such an effort.

## V. CONCLUDING REMARKS

The present paper has discussed several advances in rtMRI technology and data analysis methods, with ongoing and envisioned lines of research based on these advances. With current imaging and audio acquisition capabilities, it is possible to collect: (i) data tailored to the goals of specific linguistic studies; and (ii) large amounts of general-purpose speech production data that open up novel corpus-driven scientific research as well as technological efforts such as in automatic speech and speaker recognition. The USCTIMIT database, which consists of midsagittal rtMRI data from ten speakers who produce the 460-sentence MOCHATIMIT corpus, with complementary EMA data from four of these speakers producing the same corpus, and a collection of supporting analysis tools, has been made freely available to the research community.

Recent developments continue to increase the spatiotemporal resolution of rtMRI. The novel Sequence 4 has a temporal resolution at 12 ms, which is sufficiently fine to capture accurately fast aerodynamic events, like those in the production of trills, and latencies involved in interarticulator coordination. The nominal frame rates of Sequences 1–3 (in Table 1) are adequate for visualization of articulatory postures and movements, especially in the context of studying compositionality in space. Note that these frame rates can be increased by changing the TR-increment for view sharing down to one TR (which nevertheless does not change the time needed to acquire a full image) for better exploring compositionality in time [17]. It is also imaginable to leverage the much higher temporal resolution of EMA data, either via co-registration, or by using EMA to animate models built from rtMRI data.

RtMRI is not restricted to imaging dynamically the midsagittal slice of the vocal tract but can also image other slices of interest to the study of speech production, such as parasagittal, coronal, axial or oblique. We have recently demonstrated the possibility of acquiring, in parallel, images from multiple slices of the vocal tract [20, 35]. Our goal is to build upon the foundation of the USCTIMIT database, by adding data from slices of interest other than the midsagittal, with higher spatio-temporal resolutions, acquired from more speakers both of English and other languages, and to expand the toolset to allow for more sophisticated inspection and analysis of these data.

RtMRI for speech production research presents some shortcomings, most of which are open research topics for the USC team. First, rtMRI is currently done in a supine position, which is not a natural posture for speech, almost exclusively performed in the upright position. Much literature has been devoted to the assessment of differences in speech articulation between the two positions [74–77], and it has been suggested that the differences seem limited and that compensatory mechanisms, at least in healthy subjects, appear to be sufficiently effective to allow the acquisition of meaningful speech data in the supine position [26]. The potential use of upright, or open-type, scanners would fully remove this consideration, and there have been a few studies that demonstrate the utility of such scanners in upper-airway MRI [78, 79].

The MRI scanner is a very noisy environment, and subjects need to wear earplugs during acquisition, thus not having natural auditory feedback. Though it may be reasonable to expect that the subjects would speak much louder than normal, or that their articulation would be significantly affected as a result, it was observed on our site that, in practice, these statements held true only for rare cases of subjects. It is possible that somatosensory feedback compensates for the shortage of auditory feedback [80, 81]. Expert phoneticians that participated as subjects in rtMRI data collections at USC reported that the lack of auditory feedback presented a problem only when they tried to produce certain speech sounds *not* present in their native languages.

Because of the magnetic fields involved, people need to be excluded from being subjects in speech MRI research if they have prosthetics such as pacemakers or defibrillators, which are identified in a screening process [82]. People with a history of claustrophobia need also be excluded [83]. Otherwise, subject comfort is usually not an issue for adult healthy subjects, and for observed scan durations (overall time spent in the scanner) of less than 90 min.

Dental work is not a safety concern, but may pose problems in imaging. However, the disruptions associated with it do not consistently degrade image quality. In general, image quality is subject-dependent and in some cases it can be difficult to even maintain constant quality throughout the speech sample [84]. We have seen on our site that the impact of dental work appears to be more prominent when such work resides on the plane that is imaged, and often localized around the dental work: for example, orthodontic permanent retainers at the upper incisors result in loss of midsagittal visual information from a small circle (typically with diameter up to 3 cm) around the upper incisors.

The teeth themselves are invisible in MRI, because of their chemical composition. Various methods have been used to superimpose the teeth onto MRI images, including using data from supplementary CT imaging [85], dental casts [86, 87], or MRI data acquired using a contrast agent in the oral cavity such as blueberry juice [88] or ferric ammonium citrate [89], leaving the teeth as signal voids. Superimposing the teeth on rtMRI sequences would be useful for the exact modeling of anterior fricative consonants. At the time of writing, the data disseminated to the research community by the USC team do not include information on the teeth.

## Figures and Tables

**Table 1:** Technical details of four extensively used rtMRI sequences

	Sequence 1	Sequence 2	Sequence 3	Sequence 4

Magnetic field strength	1.5 Tesla	1.5 Tesla	1.5 Tesla	1.5 Tesla
Gradients	ZOOM	ZOOM	ZOOM	ZOOM
Spatial gradient Max. amplitude	22 mT/m	40 mT/m	40 mT/m	40 mT/m
waveform design Max. slew rate	77 T/m/s	150 T/m/s	150 T/m/s	150 T/m/s
Coil	4-channel	4-channel	4-channel	8-channel
Slice thickness	5 mm	5 mm	5 mm	6 mm
Readout duration (*T_read_*)	2.552 ms	2.520 ms	2.584 ms	2.520 ms
Repetition time (TR)	6.164 ms	6.004 ms	6.028 ms	6.004 ms
Field of view (FOV)	20 cm × 20 cm	20 cm × 20 cm	20 cm × 20 cm	20 cm × 20 cm
Spatial resolution	3.0 mm × 3.0 mm	2.4 mm × 2.4 mm	3.0 mm × 3.0 mm	2.4 mm × 2.4 mm
Pixel dimension	68 × 68	84 × 84	68 × 68	84 × 84
Number of interleaves	13	13	9	2 (Golden angle interleaving)
Relative SNR efficiency	1.00	0.63	0.83	(Not assessed)
Time to acquire full image	80.1 ms	78.1 ms	54.3 ms	12 ms
View-sharing TR-increment	7	7	5	(No view-sharing)
Reconstruction frame-rate	23.18 fps	23.79 fps	33.18 fps	83 fps
